# Reliability of the evidence to guide decision-making in the treatment of gastroesophageal reflux disease with acupuncture: protocol for an overview of systematic reviews

**DOI:** 10.1186/s13643-024-02591-4

**Published:** 2024-07-16

**Authors:** Jinke Huang, Jiali Liu, Fengyun Wang, Xudong Tang

**Affiliations:** 1 Institute of Digestive Diseases, Xiyuan Hospital of China Academy of Chinese Medical Sciences, Beijing, China; 2 Postdoctoral Research Station, China Academy of Chinese Medical Sciences, Beijing, China

**Keywords:** Gastroesophageal reflux disease, Acupuncture, Evidence, Reliability

## Abstract

**Background:**

Growing numbers of randomized clinical trials-based systematic reviews and meta-analyses (SRs/MAs) have been conducted to examine the effectiveness of acupuncture in treating gastroesophageal reflux disease (GERD). An overview of SRs/MAs will be conducted with the aim of systematically compiling, evaluating, and synthesizing the evidence regarding acupuncture for GERD.

**Methods:**

SRs/MAs of acupuncture on GERD will be searched in eight databases. Two independent reviewers will conduct the literature search, data extraction, and review quality assessment. Utilizing the AMSTAR-2 tool, PRISMA checklists, and GRADE system, respectively, the methodological quality, reporting quality, and evidence quality will be evaluated. In relation to the subject and the overview’s objects, the results will be given. This study will aid in identifying gaps between evidence and its clinical application and serve as a roadmap for further high-quality research.

**Discussion:**

The results of the overview will aid in closing the gap between clinical evidence and its use in clinical practice. This study will identify significant faults in the use of evidence, point out areas where methodology needs to be improved, and provide guidance for future high-quality research.

**Systematic review registration:**

PROSPERO CRD42022371850.

**Ethics and dissemination:**

Ethics approval is not necessary because no personal information about individuals is collected. A peer-reviewed journal or pertinent conferences will publish the results, whichever comes first.

## Background

Gastroesophageal reflux disease (GERD) is a chronic digestive disorder characterized by heartburn and regurgitation brought on by gastroesophageal reflux [1]. The chronic and highly prevalent nature of GERD confers a significant socioeconomic burden worldwide, with approximately 20% of the population in Western countries [2] affected and 5–18% of the population in Asian countries [3]. The expenditures due to GERD are enormous, amounting to $15–20 billion in the USA alone in 2006, with the major expenditures on therapeutic agents [4]. Current treatment strategies for GERD aim to alleviate or reduce gastric acid secretion, and commonly used medications include proton pump inhibitors, antacids, and histamine receptor antagonists [5]. However, the effectiveness of the available medicinal therapies varies greatly, and the majority of people need to take medication for an extended period of time or perhaps for the rest of their lives [6]. In addition, drug resistance has been observed in some patients, who in some cases have been forced to undergo surgical intervention [7]. Therefore, a better understanding of the evidence for the treatment of GERD with complementary and alternative therapies and assessment of their applicability to the management of GERD are currently urgent issues to be addressed.

Acupuncture has been used to treat gastrointestinal disorders such as GERD, and evidence on this topic is emerging. Not all systematic reviews (SRs)/meta-analyses (MAs), however, can offer trustworthy evidence, and the clinical decision-making process may be misled by poor-quality evidence [8]. Additionally, measurement tools like AMSTAR-2 [9], PRISMA [10], and GRADE system [11] were introduced in 2007, 2009, and 2004 correspondingly to assure the standardization of evidence sources.

An overview of SRs/MAs will be required with the aim of systematically compiling, evaluating, and synthesizing the evidence when several of SRs/MAs are published for related issues in a short period of time.

## Methods

### Patient and public involvement

The public and the patients will not be involved.

### Registration and protocol

The PROSPERO database has this protocol listed as registered (CRD42022371850). We will report the review in the detail mandated by the PRISMA checklists [10].

### Review eligibility criteria

#### Studies of a certain type

Randomized clinical trials examining the effects of acupuncture for GERD were enrolled by SRs/MAs. Network meta-analyses will not be included.

#### Types of participants

Regardless of sex, age, race, or illness course, participants are given a diagnosis of GERD in accordance with the internationally recognized criteria for diagnosis.

#### Types of interventions

Patients with GERD in the experimental group will receive acupuncture alone or acupuncture plus conventional medication, while patients with GERD in the control group will receive sham acupuncture or conventional medication alone.

#### Types of outcomes

Global symptom improvement will be evaluated as the primary outcome. Symptom score, quality of life, recurrence rate, and adverse events will be evaluated as the secondary outcomes.

### Search strategy

We will report search strategies in the detail mandated by the PRISMA-S extension [12]. PubMed, Web of Science, Embase, Cochrane Library, China National Knowledge Infrastructure, VIP, SinoMed, and Wanfang will be searched from their inception to August 2023. In order to locate pertinent studies, references to systematic reviews on this subject will also be checked. The specific search strategy is modified according to different databases. Table 1 provides the search strategy for the PubMed database.

**Table 1:** Search strategy for PubMed

Query	Search term
#1	Gastroesophageal Reflux [Mesh]
#2	Gastroesophageal Reflux Disease [Title/Abstract] OR Gastric Acid Reflux [Title/Abstract] OR Gastro-Oesophageal Reflux [Title/Abstract] Gastro Oesophageal Reflux [Title/Abstract] OR Esophageal Reflux [Title/Abstract] OR Gastro-Esophageal Reflux [Title/Abstract] OR Gastro Esophageal Reflux [Title/Abstract] OR Esophagitis [Title/Abstract] OR Oesophagus [Title/Abstract]
#3	#1 OR #2
#4	Acupuncture [Mesh] OR Acupuncture Therapy [Mesh]
#5	Acupuncture [Title/Abstract] OR Acupotomy [Title/Abstract] OR Acupotomies [Title/Abstract] OR Pharmacopuncture [Title/Abstract] OR Needle [Title/Abstract]
#6	#4 OR #5
#7	Meta-Analysis as Topic [Mesh] OR Systematic Reviews as Topic [Mesh]
#8	Meta-Analysis as Topic [Publication Type] OR Systematic Review [Publication Type]
#9	Systematic review [Title/Abstract] OR Meta-analysis [Title/Abstract] OR Meta analysis [Title/Abstract] OR Meta-analyses [Title/Abstract] OR Metaanalysis [Title/Abstract]
#10	#7 OR #8 OR #9
#11	#3 AND #6 AND #10

### Evaluation of eligibility and extraction of data

**Fig. 1:**
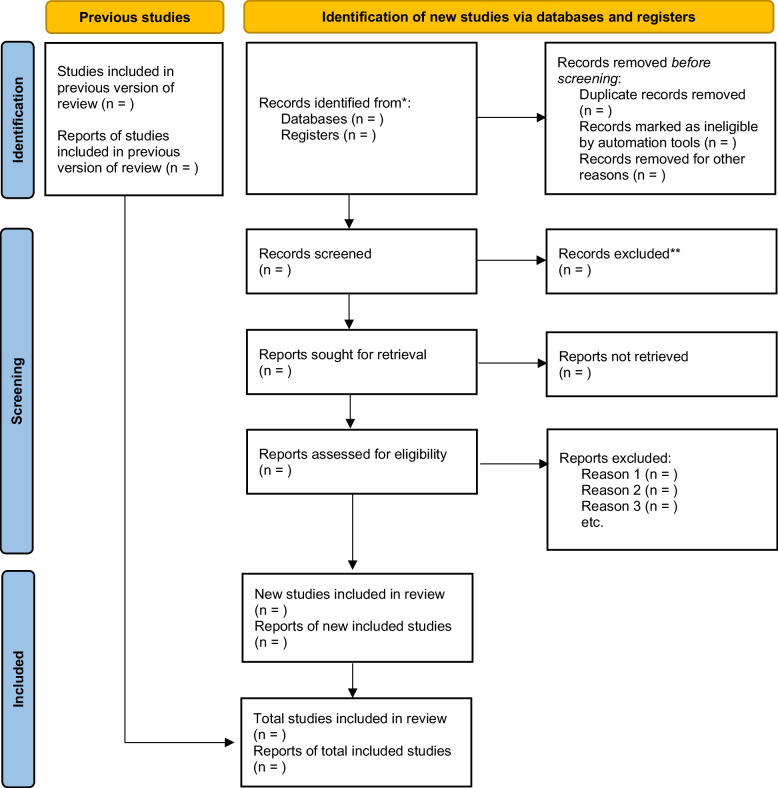
PRISMA flow chart for literature screening

By two independent reviewers, the literature will be screened (Fig. 1). A knowledgeable third reviewer will arbitrate any disagreements.

By two independent reviewers, the data from the included studies will be extracted. The initial author, publication year, nation, the number of trials that were enrolled, techniques for quality assessment, interventions, and comparisons, as well as the primary outcomes and data synthesis techniques, will be extracted. A knowledgeable third reviewer will arbitrate any disagreements.

### Quality assessment

Two independent reviewers will evaluate the methodological quality of the included reviews using the AMSTAR-2 instrument [9]. In addition, PRISMA checklists [10] will be used to evaluate the reporting quality of the included SRs/MAs. The quality of the evidence will be evaluated using the GRADE system [11] as a final step. For certainty of evidence, if the authors of the included studies have implemented an evaluation of the quality of the evidence, we will still prioritize the use of the results of our evaluations to ensure a consistent process of evaluating the certainty of the evidence across all included studies. A knowledgeable third reviewer will arbitrate any disagreements.

### Data synthesis

It will be done in an overview with a descriptive analysis [13]. Data from individual trials may have been pooled more than once in SRs/MAs included in the overview; as a result, this study will not undertake a meta-analysis. Results from the included reviews will be presented as weighted mean differences or standard mean differences with 95% confidence intervals for continuous data and as risk ratios or odds ratios for dichotomous data. Both tabular and graphical representations of the quality assessment’s findings will be provided.

## Discussion

Acupuncture has long been used clinically to treat GERD in China. In spite of this, the researchers are adamant that this treatment has not yet reached its full potential in an actual-life setting. The application of acupuncture in real-world dynamics differs from its evidence-based clinical application.

The evidence gained from SRs/MAs, which are thought of as the gold standard for evaluating the effectiveness of clinical interventions, is currently facing difficulties because of the numerous risks of bias that are created during the development of evidence [14]. Low-quality evidence may mislead decision-makers, whereas high-quality SRs/MAs can give trustworthy evidence [8]. Therefore, the veracity of the evidence and its practical implementation are not always congruent. Evidence from SRs/MAs regarding acupuncture for GERD has emerged in recent years. Nevertheless, their outcomes are inconsistent and of variable quality. The creation of clinical protocols and healthcare judgments are all jeopardized by these problems. An overview of SRs/MAs in this field is required in light of the current situation. This review’s ultimate objective is to give a full assessment of existing evidence on a variety of similar topics, to give evidence users with more targeted and superior information, and to uncover fundamental problems in evidence use [15]. The comprehensive research design for conducting an overview on the treatment of GERD with acupuncture is described in this protocol. By making this protocol public, we can assure that its overview may be reproduced by others and gain insight from the comments of subject matter experts. However, limitations of this study need to be acknowledged as MEDLINE (Medical Literature Database Series) will not be accessible, which may lead to bias.
